# Robust Feature Matching for 3D Point Clouds with Progressive Consistency Voting

**DOI:** 10.3390/s22207718

**Published:** 2022-10-11

**Authors:** Siwen Quan, Kunpeng Yin, Kaixiao Ye, Kechen Nan

**Affiliations:** School of Electronics and Control Engineering, Chang’an University, Xi’an 710064, China

**Keywords:** 3D point cloud, feature matching, progressive consistency voting, geometric constraints, point cloud registration

## Abstract

Feature matching for 3D point clouds is a fundamental yet challenging problem in remote sensing and 3D computer vision. However, due to a number of nuisances, the initial feature correspondences generated by matching local keypoint descriptors may contain many outliers (incorrect correspondences). To remove outliers, this paper presents a robust method called progressive consistency voting (PCV). PCV aims at assigning a reliable confidence score to each correspondence such that reasonable correspondences can be achieved by simply finding top-scored ones. To compute the confidence score, we suggest fully utilizing the geometric consistency cue between correspondences and propose a voting-based scheme. In addition, we progressively mine convincing voters from the initial correspondence set and optimize the scoring result by considering top-scored correspondences at the last iteration. Experiments on several standard datasets verify that PCV outperforms five state-of-the-art methods under almost all tested conditions and is robust to noise, data decimation, clutter, occlusion, and data modality change. We also apply PCV to point cloud registration and show that it can significantly improve the registration performance.

## 1. Introduction

Feature matching for 3D point clouds is a fundamental problem in remote sensing and 3D computer vision with a number of applications such as point cloud registration [1,2,3,4,5], 3D object recognition [6], and localization [7]. Its objective is to establish reasonable point-to-point correspondences between two given point clouds (this paper focuses on 3D rigid point clouds). Usually, such correspondences are a subset of initial feature correspondences with outliers (incorrect correspondences) generated by matching local keypoint descriptors [8,9,10]. Therefore, the key issue to robust feature matching for point clouds is how to distinguish inliers from outliers. This task is particularly challenging when the initial correspondence set is contaminated by severe outliers [11,12], which is generally caused by the following factors. **(1)** *Keypoint detection errors*. The repeatability of existing 3D keypoint detectors [13] is limited to real-world data, especially for data captured by low-cost sensors. In this case, some truly corresponding points cannot be discovered. **(2)** *Limited descriptiveness of 3D local descriptors*. The premise for correct correspondences is that the similarity of two local surface patches can be reasonably estimated, which is accomplished by 3D local descriptors. However, most of the existing 3D local descriptors show limited descriptiveness in the presence of partial overlap, clutter, and occlusion [14]. **(3)** *Data nuisances*. Due to the limitations of current 3D acquisition systems, the raw captured point clouds are usually quite noisy. Variation of the distance from the sensor to the object/scene will further result in varying data resolutions. In addition, complex scenes with self-occlusion, occlusion, and clutter may lead to point clouds with incomplete or redundant data. These data nuisances further possess great challenges to robust point cloud correspondences.

To overcome the above difficulties, many attempts have been done by researchers in the past decade. Some methods select candidates from the initial correspondence set solely using the feature similarity cue, e.g., correspondence selection based on the nearest neighbor (NN) [15,16] or the nearest neighbor similarity ratio (NNSR) [17,18]. Although simple and fast, methods relying on feature similarity are very sensitive to outliers because the distinctiveness of local descriptors cannot be guaranteed on challenging data. The popular estimator random sample consensus (RANSAC) [19] can also be employed for outlier rejection [2,20] in the context of point cloud feature correspondences. Nonetheless, it is not appropriate to directly apply RANSAC or its variants to find consistent correspondences from the initial set because these estimators usually require a huge amount of repetitions and may still fail to find the optimal solution. A more reasonable way, as suggested by many studies [21,22], is to first score feature correspondences and then rank them to screen out top-scored correspondences as the final feature matching result. Some advances have been made along this research line, e.g., the spectral technique (ST) [23] and search of inliers (SI) [21]. Note that NN and NNSR can be also categorized as correspondence scoring techniques since the similarity of two keypoints can be served as the confidence score as well. Unfortunately, as revealed by a recent evaluation study [11], the performance of existing scoring methods is still limited when faced with severe outliers.

Under these considerations, we propose a novel scoring technique named progressive consistency voting (PCV) to achieve robust feature matching for 3D point clouds. Given a set of initial correspondences generated by matching local keypoint descriptors, PCV assigns a confidence score to each correspondence. Accordingly, a subset of high-quality correspondences, i.e., the expected output of feature matching, can be screened out by simply considering top-scored candidates. The basic idea of PCV is finding a reliable voting set from the initial set and aggregating the geometric consistencies between the correspondence to be scored and these candidates in the voting set under a voting scheme. Although some voting-based scoring methods already exist [21,22], they underestimate the significance of the voting set, which is critical to achieve robust feature matching, as will be verified in Section 4.4. By contrast, the proposed PCV, to the best of our knowledge, is the first method that highlights the issue of mining reliable voters and optimizes the voting set in a progressive manner. Moreover, unlike previous voting-based methods needing special care for the design of geometric constraints, PCV manages to achieve decent performance using very simple geometric constraints. Experiments on several public datasets verify that PCV is robust to a number of nuisances. A comparison with six state-of-the-art methods further confirms the overall superiority of the proposed method. In a nutshell, this paper presents three main contributions.

- We propose a feature matching method PCV for 3D point clouds, which progressively optimizes a voting set and the confidence scores of correspondences based on the geometric consistencies between correspondences. Experiments and comparison with state-of-the-art methods demonstrate that PCV is robust to noise, data decimation, clutter, occlusion, and data modality change. Moreover, PCV can achieve outstanding performance with very simple geometric constraints.
- PCV highlights and demonstrates the significance of the voting set to robust 3D feature matching for voting-based methods, which may inspire other related methods.
- We show that PCV can be successfully applied to point cloud registration and improve the registration performance under existing pipelines.

The remainder of this paper is organized as follows. Section 2 presents a brief overview of existing 3D feature matching materials and methods. Section 3 elaborates the proposed PCV method. Section 4 presents the experimental results on several standard datasets and the comparative results with some state-of-the-art methods. Finally, Section 5 draws the conclusions.

## 2. Related Works

Existing feature matching methods for point clouds tend to assign confidence scores to feature correspondences. There also exist some methods that assign binary labels (i.e., true or false) to correspondences.

### 2.1. Methods Computing Binary Labels

RANSAC [19] distinguishes inliers from outliers based on a “hypothesis generation and verification” mechanism; three or more correspondences are first randomly sampled from the initial correspondence set; a hypothesis is then generated with these samples and candidates agreeing the hypothesis are judged as inliers at this iteration; the above processes are repeated, and the inlier set with the maximum cardinality is served as the final result. Some variants of RANSAC have also been proposed in 3D domain, e.g., the optimal sample consensus (OSAC) [15], 1-point RANSAC (1P-RANSAC) [6], RANSAC with different inlier metrics [24]. A recent comprehensive evaluation on RANSAC methods for 3D correspondence grouping and registration can be referred to [25]. However, the RANSAC family generally holds limited scalability and can hardly find the optimal solution when confronted with massive outliers [11]. Chen and Bhanu [26] grouped correspondences with a geometric consistency (GC) framework; they measured the consistency score between any two correspondences and formed a cluster for each correspondence with its compatible ones; the maximum cluster is treated as the inlier cluster. Tombari and Stefano [27] presented a 3D Hough voting (3DHV) approach that projects each correspondence to a 3D Hough space using the local reference frame (LRF) of each keypoint; projected points forming a cluster in the Hough space are identified as inliers. Ma et al. proposed robust hypothesize-and-verify methods that rely on vector field consensus (VFC) [28] and robust point matching via $L_{2}E$ [29], respectively. Lu et al. [30] employed redundant geometric constraints to determine the best transformation and correspondences agreeing with the best transformation are grouped as inliers. More recently, Ma et al. [31] and Zhao et al. [32] cast the feature matching problem into a two-class classification problem and learned a general classifier to determine the correctness of a correspondence. In order to maintain the local neighborhood structures of potential inliers, Ma et al. [33] proposed a locality preserving matching (LPM) model to identify correct correspondences.

The above methods are either parametric (e.g., RANSAC) or non-parametric (e.g., GC and 3DHV). Parametric methods cannot scale well to large-scale feature matching problems and are sensitive to outliers; the precision performance of many non-parametric methods remains limited [11].

### 2.2. Methods Computing Confidence Scores

A straightforward correspondence scoring approach is serving the feature similarity between the two associated keypoints of a correspondence as the confidence score [15,34]. Typically, $L_{2}$ distance is employed as the distance metric. Rusu et al. [35] and Yang et al. [16] investigated the effect when using other different distance metrics on point cloud feature matching. Because local descriptors are sensitive to repetitive patterns, Lowe [17] presented a nearest neighbor similarity ratio (NNSR) method to prefer distinctive correspondences. Unfortunately, purely leveraging the feature similarity cue inherits the sensitivity of the employed local descriptor to some certain nuisances such as clutter and occlusion.

Compared with feature similarity, geometric constraints are more robust cues. Leordeanu and Hebert [23] introduced a spectral technique (ST) to recover inliers from initial correspondences through analyzing the adjacency matrix of a graph with each node being a correspondence and each edge being the pairwise consistency of two nodes. Rodolà et al. [36] selected correspondences that satisfy global geometric consistency constraints with a game theory matching (GTM) model. Cirujeda et al. [37] presented a variant of GTM by adding feature constraints to the pay-off function. A limitation of GTM-based methods is that they are highly selective, thus failing to strike a good balance between precision and recall. Buch et al. [21] proposed finding inliers by local and global voting; the local voting stage checks the geometric consistency of a correspondence with its neighbors via a distance constraint; the global stage judges the global geometric consistency using an LRF constraint. Following the voting scheme, Yang et al. [22] proposed a consistency voting (CV) approach that treats distinctive correspondences as the voting set and assign voting scores to correspondences based on the rigidity and LRF constraints. Note that although the proposed PCV method also follows a voting fashion like SI and CV, it holds some unique characteristics. **(1)** SI and CV ignore the significance of the definition of voting set, and they regard either spatially close or distinctive correspondences as voters. This cannot guarantee the quality of the voting set and fails to assign reasonable voting scores to correspondence as will be verified in Section 4.4. By contrast, our PCV highlights the significance of the voting set and progressively optimizes the voting set. **(2)** Unlike SI and CV needing carefully designed geometric constraints, the proposed PCV method is able to achieve the state-of-the-art performance with a simple distance constraint.

## 3. Methods

**Figure 1:**
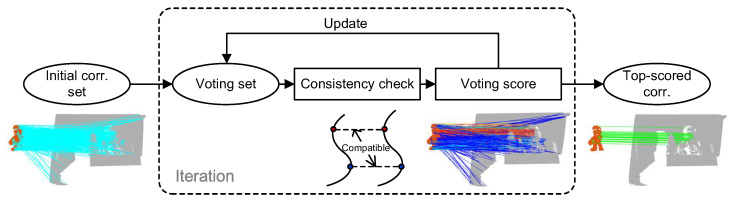
Pipeline of the proposed PCV method. Given initial correspondences (cyan lines), PCV iteratively updates the voting set, checks the geometric conistency of each correspondence with the voters in the voting set, and assigns a voting/confidence score to each correspondence (rendered in colored lines). Consistent correspondences (green lines) can be found by simply ranking the correspondences based on voting scores.

This section elaborates the technique details of the proposed PCV method (Figure 1). PCV iteratively refines the confidence score assigned to each correspondence. Each iteration contains three main parts, i.e., definition/update of the voting set, pairwise compatibility assessment, and voting score calculation.

### 3.1. Notations and Problem Formulation

Let $P^{s}$, $P^{t}$, and $C_{initial}$ be the source point cloud, target point cloud, and the initial correspondence set between them, respectively. $C_{initial}$ is usually generally obtained by matching local keypoint geometric descriptors such as the signature of histograms of orientation (SHOT) [38] and local feature statistics histograms (LFSH) [15]. A candidate in $C_{initial}$ can be parametrized as $c=(p^{s},\;p^{t},\;s_{f}\left(p^{s},\;p^{t}\right))$, where $p^{s}$ and $p^{t}$, respectively, are two keypoints in $P_{s}$ and $P^{t}$, and $s_{f}\left(p^{s},\;p^{t}\right)$ is the feature matching score of $p^{s}$ and $p^{t}$. By default, we use Lowe’s ratio technique [17] to assign $s_{f}\left(p^{s},\;p^{t}\right)$ to *c*.

The objective is to assign a confidence score s(c) to each correspondence. Accordingly, an inlier set $C_{inlier}$ can be efficiently calculated by finding top-scored candidates in $C_{initial}$.

### 3.2. Pairwise Compatibility Assessment

The proposed method iteratively checks the compatibility of each correspondence in $C_{initial}$ and all candidates in a predefined voting set $C_{voting}$. The motivation behind is that, if a correspondence is correct, it will receive a high voting score if most of the candidates in the voting set are correct as well, because only correct correspondences are geometrically compatible with each other [11]. Therefore, the calculation of the voting score relies on the assessment of the pairwise compatibility of two correspondences. The definition and update of the voting set $C_{voting}$ will be detailed in Section 3.4, and in the following we assume that $C_{voting}$ has been given.

**Figure 2:**
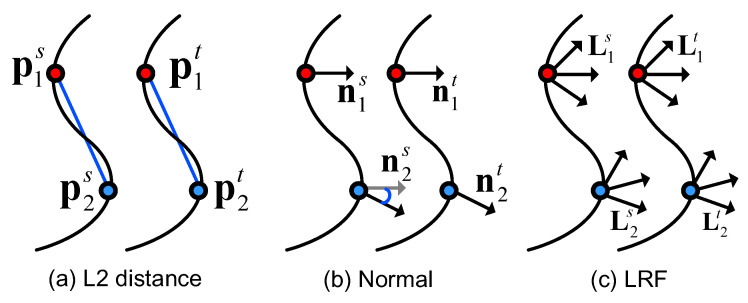
Three popular geometric constraints for the assessment of correspondence compatibility (illustrated in 2D).

Now, the first question is how to measure the compatibility of two correspondences $c_{1}$ and $c_{2}$. In the literature, many effective geometric constraints have been proposed for the assessment of correspondence compatibility, where $L_{2}$ distance [21,36], normal [39,40], and LRF [21,22] are three typical ones. To better understand and compare the three constraints, we give a schematic illustration for them in Figure 2.

The rigidity constraint (Figure 2a) is defined as:

(1) $$E_{L_{2}}=|∥p_{1}^{s}-p_{2}^{s}∥-∥p_{1}^{t}-p_{2}^{t}∥|.$$

The normal constraint (Figure 2b) is defined as:

(2) $$E_{normal}=\left|\mathrm{acos}\left(n_{1}^{s}\cdot n_{2}^{s}\right)-\mathrm{acos}\left(n_{1}^{t}\cdot n_{2}^{t}\right)\right|.$$

where n is the normal vector of p. Let L be the LRF at p. LRF is a local canonical frame established at keypoint p to make the local keypoint descriptor rotation invariant. An LRF can be mathematically represented by a 3×3 matrix constituted by three orthogonal unit vectors. The LRF constraint (Figure 2c) is defined as:

(3) $$E_{lrf}=\left|l_{diff}^{s}-l_{diff}^{t}\right|,$$

where

(4) $$l_{diff}=\mathrm{acos}\left(\frac{\mathrm{trace}(L_{1}{L_{2}}^{-1})-1}{2}\right)\frac{180}{\pi }.$$

Most of existing methods [21,22,40] make great efforts to either carefully combine above geometric constraints or devise new ones. By contrast, our method, with progressively optimized voters, can achieve decent performance with the simple $L_{2}$ distance constraint, which is quite efficient for calculation (as will be verified in Section 4.4). Note that the $L_{2}$ distance constraint only needs keypoint locations, which are already provided at the keypoint detection stage, while other constraints such as normal and LRF need additional computational costs as well as efforts on tuning associated parameters. Therefore, we define the compatibility score $F(c_{1},\;c_{2})$ between $c_{1}$ and $c_{2}$ as:

(5) $$F\left(c_{1},\;c_{2}\right)=\exp\left(-\frac{{E_{L_{2}}}^{2}}{2\tau^{2}}\right),$$

where τ is a distance parameter. Based on empirical tests, τ is set to 10 *pr*. The unit *pr*, here and hereafter, denotes the point cloud resolution, i.e., the average shortest distance between each point and its neighbors in a point cloud, which can be mathematically defined as:

(6) $$pr=\frac{1}{\left|P\right|}\sum_{p_{i}\in P}\left|\right|p_{i}-p_{nn}\left|\right|,$$

where $p_{i}$ represents a generic point in point cloud P and $p_{nn}$ is the nearest neighbor of $p_{i}$.

### 3.3. Voting Score Calculation

At each iteration of PCV, e.g., the *i*th iteration, a voting score $s_{i}\left(c\right)$ will be assigned to each correspondence *c*. $s_{i}\left(c\right)$ is the integration of the compatibility scores of *c* with each candidate in the voting set $C_{voting}^{i}$ at the current iteration. More specifically, $s_{i}\left(c\right)$ is defined as:

(7) $$s_{i}\left(c\right)=\sum_{c_{j}\in C_{voting}^{i}}F(c,\;c_{j}).$$

From the above equation, one can find that the voting score is independent from feature similarities that are sensitive to clutter, occlusion, and limited overlap. We repeat the voting score calculation process for $N_{iter}$ times and serve the voting scores at the last iteration as the final scoring result.

### 3.4. Definition and Update of the Voting Set

The definition and update of the voting set is a peculiarity of the proposed PCV method and acts as a critical component. The basic goal is to find a subset from $C_{initial}$ with a high inlier ratio as the voting set $C_{voting}$, so the voters are more convincing and the voting scores are more distinguishable. However, the label of each correspondence is unknown and in fact it is the objective of feature matching. To solve this problem, we propose the following method to progressively optimize the voting set.

At the first iteration of PCV, we define the voting set as the collection of correspondences with top $s_{f}\left(p^{s},\;p^{t}\right)$ values, denoted by $C_{voting}^{0}$. Remarkably, the definition $C_{voting}^{0}$ has a relatively slight impact on the performance of PCV. PCV is even able to achieve good performance with $C_{voting}^{0}$ composed of randomly selected correspondences (as will be verified in Section 4.4).

Because $s_{f}\left(p^{s},\;p^{t}\right)$ is sensitive to clutter, occlusion, and limited overlap, $C_{voting}^{0}$ may contain many outliers. To optimize $C_{voting}^{0}$, we suggest updating $C_{voting}$ based on the scoring result of the previous iteration. This is reasonable because [22] has verified that the confidence scores of correspondences can be optimized within the consistency voting framework. Moreover, we experimentally observed that the quality of the correspondence scoring result generally improves during iterations (as will be shown in Section 4.4). As such, $C_{voting}^{i}$ at the *i*th iteration of PCV can be updated as follows:

(8) $$C_{voting}^{i}={\left\{c_{j}\in C_{initial}^{i-1}\right\}}_{j=1}^{\kappa },$$

where $C_{initial}^{i-1}$ is the sorted $C_{initial}$ according to the scoring result (Equation (7)) in a descending order at the previous iteration and κ is the cardinality of $C_{voting}^{i}$.

**Figure 3:**
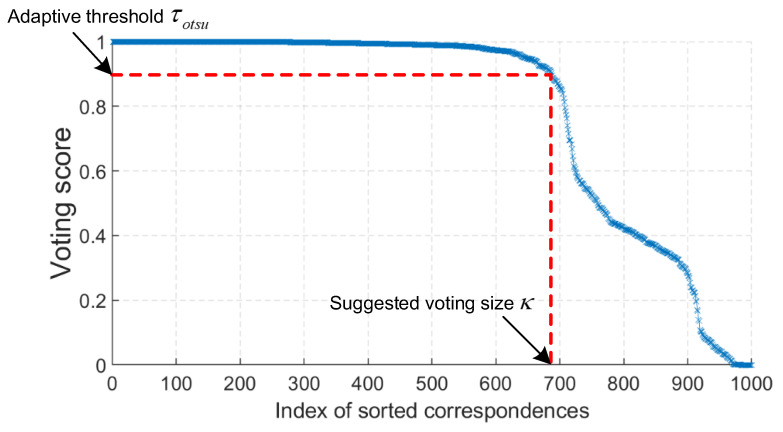
An adaptive strategy to determine the cardinality of the voting set. $C_{initial}$ is first sorted according to the voting scores at the i−1th iteration; an adaptive threshold $\tau_{otsu}$ is then computed based on Otsu’s thresholding method [41]; the number of correspondences whose voting scores are greater than $\tau_{otsu}$ is defined as κ. The point cloud pair used to generate this figure is taken from the Bologna Dataset1 (BoD1) [42] dataset.

The parameter κ requires special attention, because the objective is to bring as many inliers as possible in $C_{voting}^{i}$ while rejecting outliers. If we fix κ, small values of κ will fail to include all potential inliers and large values of κ will drag in outliers if the number of inliers in $C_{initial}$ is smaller than κ. To overcome this problem, we propose a method to make κ adaptive. Specifically, we apply Otsu’s thresholding method [41] on $C_{initial}^{i-1}$ to obtain an adaptive threshold $\tau_{otsu}$. Then, we define κ as the number of correspondences whose voting scores are greater than $\tau_{otsu}$. The reason behind is that the voting scores calculated using geometric constraints hold certain discriminatory power, which is also demonstrated in Figure 3.

Therefore, the voting set in PCV on one hand is progressively optimized using the scoring result at the previous iteration and on the other has an adaptive cardinality to cope with initial correspondence sets with different scales and inlier ratios.

### 3.5. Method Summarization

Algorithm 1 summarizes the key steps of the proposed PCV method. Remarkably, once voting scores have been assigned to correspondences by PCV, we can define inliers in two ways. One is setting a threshold based on Otsu’s thresholding method [41] to split the correspondence set by comparing voting scores with the threshold. The other is simply serving *K* of top-scored correspondences as inliers, where *K* is an empirical threshold depending on application scenarios.
**Algorithm 1** Feature matching for point clouds based on PCV.**Require:** The source point cloud $P^{s}$ and the target point cloud $P^{t}$.
**Ensure:** Consistent feature correspondences $C_{inlier}$ between $P^{s}$ and $P^{t}$.
 1: Generate $C_{initial}$ by matching local keypoint descriptors;
 2: Initialize the iteration index i=0 and $C_{inlier}=Ø;$
 3: Define the initial voting set $C_{voting}^{0}$ based on $s_{f}\left(p^{s},\;p^{t}\right)$;
 4: **while**
$\;i<N_{iter}\;$
**do**
 5:    Calculate the compatibility score between $c\in C_{initial}$ and $c_{j}\in C_{voting}^{i-1}$ using Equation (5);
 6:    Calculate the voting score for $c\in C_{initial}$ using Equation (7);
 7:    Update $C_{voting}^{i}$ using Equation (8);
 8:    i=i+1;
 9: **end while**
 10: Sort $C_{initial}$ based on the scoring result at the last iteration;
 11: Push top-scored correspondences into $C_{inlier}$;
 12: Return $C_{inlier}$.


## 4. Experiments

### 4.1. Datasets

**Figure 4:**
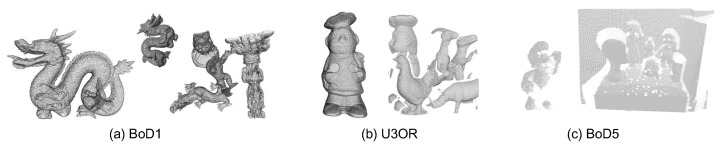
Visualization of three sample point cloud pairs from the experimental datasets.

The experimental datasets include the Bologna Dataset1 (BoD1) dataset [42], the UWA 3D object recognition (U3OR) dataset [43], and the Bologna Dataset5 (BoD5) dataset [42]. BoD1 is a synthetic recognition dataset consisting of six models and 45 scenes, where the scenes were generated by randomly rotating three to five models to create clutter and pose variations. U3OR is a renowned benchmark in 3D computer vision [14]. It has five models and 50 real scenes. This dataset also provides the quantized information about clutter and occlusion. Both BoD1 and U3OR are composed of LiDAR-scanned point clouds, while BoD5 is obtained via a Kinect sensor. BoD5 contains 43 matching pairs with severe real noise. Some sample views of the point clouds in these datasets are presented in Figure 4.

### 4.2. Criterion

Following [22,44], we employ the recall of inlier (ROI) metric to evaluate the quality of a point cloud feature matching method. The definition is given as follows.

Let $C_{inlier}^{K}$ be the set of *K* top-scored correspondences computed by a correspondence scoring method, the recall respecting a given *K* is defined as:

(9) $$recall_{K}=\frac{\#\;\mathrm{inliers}\;\mathrm{in}\;C_{inlier}^{K}}{\#\;\mathrm{inliers}\;\mathrm{in}\;C_{initial}}.$$

By varying *K*, a curve can be generated. To judge if a correspondence $c=(p^{s},\;p^{t})$ is an inlier, we use the ground truth rotation matrix $R_{gt}$ and translation vector $t_{gt}$ and check if $∥R_{gt}p^{s}+t_{gt}-p^{t}∥$ is smaller than a distance threshold $\tau_{inlier}$. We set $\tau_{inlier}$ to 5 *pr* in the experiments.

### 4.3. Compared Methods and Implementation Details

Five methods are considered for comparative evaluation, including the nearest neighbor (NN) [2,15], nearest neighbor similarity ratio (NNSR) [17], spectral technique (ST) [23], search of inliers (SI), and consistency voting (CV) [22]. NN and NNSR are served as two baselines purely rely on the feature similarity cue; ST is a well-known graph-based method for feature matching; SI and CV are two recent methods based on the voting scheme as well. The parameters of all compared methods are kept identical to the settings in the original papers.

To generate the initial correspondence set $C_{initial}$, we detect keypoints on point clouds via the Harris 3D [45] detector and use the SHOT [38] descriptor to perform local feature description. Then, we perform brute force feature matching based on the $L_{2}$ distance metric to generate $C_{initial}$. Note that the we are not aiming at finding the best combination of detector and descriptor to generate high quality $C_{initial}$. Instead, we simply choose a combination of popular detectors and descriptors to obtain initial correspondences with various qualities when confronted with different challenges. This is necessary for a comprehensive evaluation of feature matching methods.

### 4.4. Method Analysis

Before evaluating PCV’s overall performance, we are amenable to validate the effectiveness of some key components of it, to make PCV explainable. Some key parameters also need to be experimentally analyzed. We conduct these experiments on a “tuning” BoD1 dataset where the scenes are first down-sampled to $\frac{1}{4}$ of the original resolution and then injected with 0.3 *pr* Gaussian noise.

**Figure 5:**
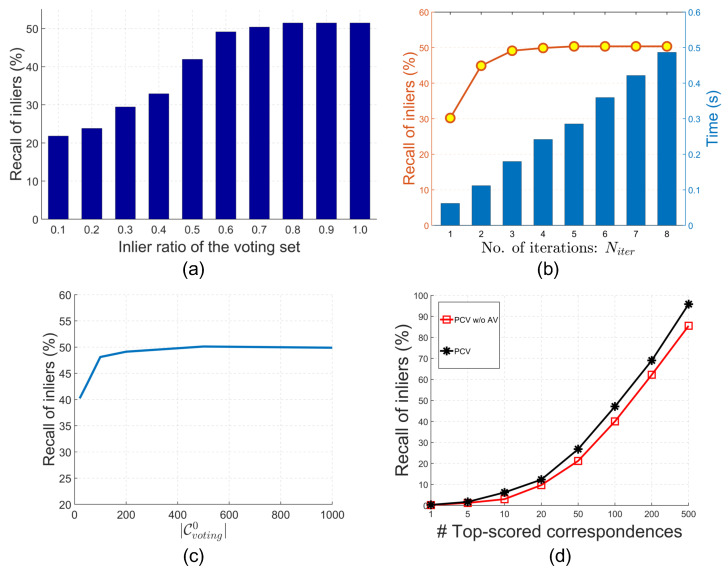
Experimental analysis for PCV. (**a**) The effect when using voting sets with different inlier ratios on the feature matching performance. (**b**) Parameter analysis for $N_{iter}$. (**c**) Parameter analysis for $|C_{voting}^{0}|$. (**d**) The effectiveness of making the cardinality of the voting set κ (Equation (8)) adaptive.

**The significance of high-quality voting set.** To demonstrate the significance of the voting set to the final feature matching performance, we generate voting sets with different inlier ratios, i.e., 0.1 to 1.0 with a gap of 0.1, and perform consistency voting to compute voting scores. Figure 5a presents the ROI performance (K=100) when using voting sets with various inlier ratios. Clearly, one can find that more inliers in the voting set result in better feature matching performance. This validates the rationality of our motivation to pursue high-quality voters.
**How many iterations are required?** PCV progressively optimizes the voting set and voting scores. Since more iterations would result in more time consumption, we should determine a proper $N_{iter}$ to achieve a trade-off between time efficiency and ROI performance. Figure 5b reports the efficiency and ROI results (K=100) when varying $N_{iter}$ from 1 to 8. It suggests that the ROI performance generally improves as $N_{iter}$ increases. This also indicates the effectiveness of the proposed progressive voting framework because more iterations achieve better performance. When $N_{iter}$ is greater than 2, the performance remains almost stable. Thus, we set $N_{iter}$ to 3 in this paper.
**The definition of $C_{voting}^{0}$.** By default, we use Lowe’s ratio rule [17] (denoted by NNSR) to determine the initial voting set $C_{voting}^{0}$. In this experiment, initial voting sets determined by selecting correspondences with top-ranked feature similarity values (denoted by NN) and random selection in $C_{initial}$ are also taken into consideration. Table 1 presents the results. It suggests that NNSR-based $C_{voting}^{0}$ achieves the best performance, possibly owing to the fact that more inliers are included in $C_{voting}^{0}$ when sorting $C_{initial}$ based on NNSR. However, using randomly selected correspondences as $C_{voting}^{0}$ also achieves promising result. We can deduce that PCV is able to effectively refine $C_{voting}$ in the subsequent iterations even with poor initializations.
**The cardinality of $C_{voting}^{0}$.** Although we have proposed an approach to make the cardinality $C_{voting}$ adaptive during the iterations, the cardinality of the initial voting set $C_{voting}^{0}$ should be pre-defined. To examine its influence, we vary $C_{voting}^{0}$ from 20 to 1000 and compute the ROI results. As shown in Figure 5c, we can see that the ROI performance (K=100) with respect to different values of $C_{voting}^{0}$ is generally stable. The only salient improvement can be found when $C_{voting}^{0}$ increases from 20 to 100. It is possibly due to the fact that few inliers are included with small $|C_{voting}^{0}|$. Because PCV updates $|C_{voting}^{i}|$ adaptively at the subsequent iterations (i.e., i>0), PCV is not very sensitive to the parameter $|C_{voting}^{0}|$ as long as it is not extremely small.
**The effectiveness of making κ adaptive.** During the iterations of PCV, we make the cardinality of the voting set κ (Equation (8)) adaptive, because fixing it can hardly guarantee the inlier ratio of the voting set. For instance, if there are 100 inliers in the initial correspondence set and we fix κ to 200, the highest inlier ratio of the voting set is 0.5. However, we have already shown that voting sets with higher inlier ratios can achieve better performance in Figure 5a. Therefore, we compare the performance of the standard PCV and PCV without adaptive voting sets (denoted by “PCV w/o AV”). The result is shown in Figure 5d. Obviously, better performance is achieved with adaptive voting sets.
**The selection of geometric constraints.** Geometric constraints are employed to measure the compatibility score between correspondences. We examine the performance of PCV when using different geometric constraints (the combinations of constraints in Equations (1)–(3)). Specifically, the $L_{2}$ distance, $L_{2}$ distance+normal, and $L_{2}$ distance+LRF constraints have been employed in existing studies [21,22,36,40]. We also consider the $L_{2}$ distance+normal+LRF constraint in this experiment, generating a total of four different geometric constraints. The results are reported in Table 2.

It shows that PCV is not sensitive to the selection of geometric constraints, since almost comparable performance is achieved when using tested constraints. Moreover, it achieves outstanding performance with the simple $L_{2}$ distance constraint. Note that other constraints such as normal and LRF need additional time consumption as well as efforts for parameter tuning. By contrast, PCV allows the use of a simple distance constraint that only requires the coordinate information of keypoints. It is potentially because PCV can mine high-quality voters and thus does not have strict demands on the selection of geometric constraints. This makes PCV quite flexible. In particular, normals and LRFs are usually computed by feature descriptors, and the time costs of employing the above different constraints are identical.

### 4.5. Feature Matching Performance

The feature matching performance of PCV and the compared methods is evaluated under various challenging conditions, including Gaussian noise, data decimation, clutter, occlusion, and data modality change.

**Figure 6:**
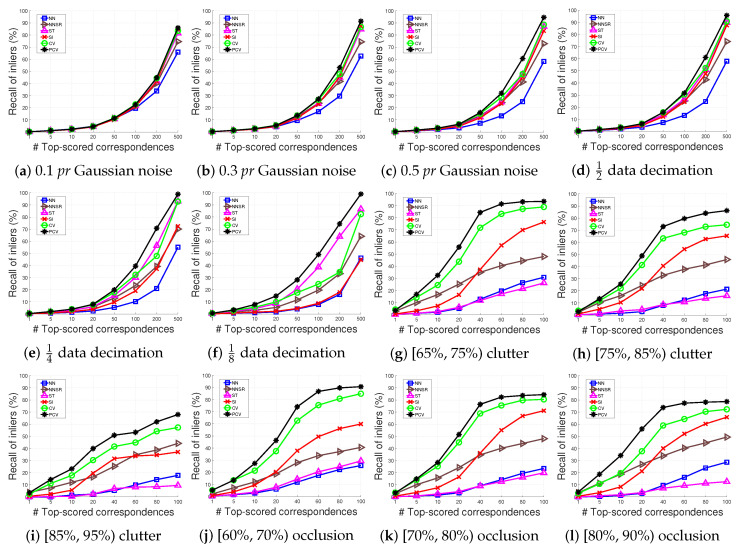
ROI performance of PCV and compared methods under different levels of (**a**–**c**) Gaussian noise, (**d**–**f**) data decimation, (**g**–**i**) clutter, and (**j**–**l**) occlusion.

**Robustness w.r.t. Gaussian noise.** We add Gaussian noise with 0.1 *pr*, 0.3 *pr*, and 0.5 *pr* standard deviations to the scenes of the BoD1 dataset, respectively. The results are shown in Figure 6a–c.

With 0.1 *pr* Gaussian noise, all tested methods generally behave comparably and our PCV is slightly better than others. As the standard deviation of Gaussian noise increases, PCV achieves the best performance and the gap between it and other compared methods is more clear. Besides, NN and NNSR are inferior to others, verifying our statement that methods simply relying on the feature similarity are not robust to some common nuisances. CV, ST, and SI deliver similar performance in this case.

**Robustness with reference to data decimation.** To assess a method’s robustness to data decimation, we down-sample the scenes of the BoD1 dataset to $\frac{1}{2}$, $\frac{1}{4}$, and $\frac{1}{8}$ of their original resolutions. The results are presented in Figure 6d–f.

Compared with Gaussian noise, data decimation has a more severe impact on feature matching. Even though, PCV achieves the best performance under all levels of data decimation. The CV method that ranks the second with $\frac{1}{2}$ data decimation meets a clear performance deterioration as the point clouds are further down-sampled. This is due to two factors. First, CV relies on the LRF constraint, and the calculation of LRF is sensitive to data decimation [38], thus affecting the calculation of compatibility scores. Second, CV simply defines the voting set based on feature similarities, as the matching case becomes challenging, the voting set contains less inliers and results in unconvincing voting scores. Overall, ST appears to be the second best method when faced with data decimation.

**Robustness with reference to clutter and occlusion.** The U3OR dataset provides the quantitative information of clutter and occlusion for each matching pair. We first split the matching pairs into three groups with [65%, 75%), [75%, 85%), and [85%, 95%) clutter, respectively. Analogously, we then generate another three groups with [60%, 70%), [70%, 80%), and [80%, 90%) occlusion, respectively. The results are shown in Figure 6g–l.

From these figures, one can see that PCV outperforms all compared methods under all levels of clutter and occlusion, followed by CV. Although CV and SI are also voting-based methods, they require more complex geometric constraints and are still inferior to PCV. It also reflects the effectiveness of the proposed progressive scheme for voting set optimization. Another remarkable phenomenon is the performance of ST. When faced with clutter and occlusion, ST is even inferior to NN and is the bottom-placed method. This is because ST has an assumption that inliers should appear in a cluster form, which is of rare occurrence when the point clouds are contaminated by severe clutter and occlusion. Generally, three voting-based methods, i.e., PCV, CV, and SI, are more robust to clutter and occlusion than other tested methods. Because voting-based methods search inliers individually, some isolated inliers may be identified.

**Figure 7:**
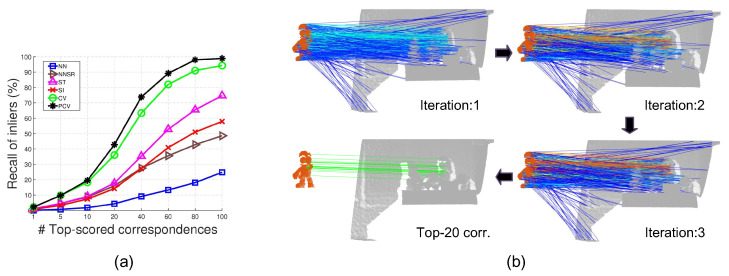
Feature matching results on the BoD5 dataset. (**a**) The ROI performance of six tested methods. (**b**) Visualization of the scoring result at each iteration of PCV (voting scores are encoded with pseudo-color).

**Robustness with reference to data modality change.** Both BoD1 and U3OR are LiDAR datasets. To test a method’s robustness to data modality change, we conduct experiments on the Kinect-captured BoD5 dataset. The results are shown in Figure 7.

As witnessed by Figure 7a, PCV consistently surpasses other compared methods on this dataset, showing strong resilience to data modality variation. We also present some visual results of PCV in Figure 7b. One can see that the confidence scores assigned to correspondences are iteratively optimized. More specifically, voting scores become more distinguishable as rendered by pseudo-color lines. Therefore, consistent correspondences can be found by ranking correspondences based on PCV’s confidence scores.

**Correspondence grouping performance.** Because one can also assign binary labels to correspondences based on PCV, we test the correspondence grouping (mismatch removal) performance of feature matchers on the whole U3OR dataset. The objective of correspondence grouping is to split the initial correspondence set into two subsets composed by inliers and outliers, respectively [11]. This experiment additionally takes the recent LPM [33] matcher into consideration. Except for LPM, other tested methods (confidence score-based ones) group correspondences based on Otsu’s thresholding strategy [41] as in [21]. We evaluate the correspondence grouping performance via precision, recall, and F-score metrics [11]. The results are shown in Table 3.

In terms of the F-score performance, the proposed PCV is the best competitor, followed by CV and LPM. In addition, PCV delivers the best precision performance and the second best recall performance. The comparison between PCV and CV again verifies the rationality of mining good voters. PCV also achieves better performance than LPM. A potential reason is that PCV defines the correctness of correspondences based on global geometric consistencies while LPM prefers correspondences preserving local structures. In particular, regarding the recall performance, PCV is slightly worse than LPM. This is because PCV is an iterative method, which imposes tighter constraints to correspondences than the non-iterative LPM method. For point clouds undergoing a rigid transformation model, global consistencies are supposed to be more reliable.

In general, it is very difficult to achieve good performance under a variety of nuisances and we can find the performance of most compared methods fluctuates significantly under different nuisances. However, PCV consistently achieves the best performance under all tested conditions. It can be explained from at least two aspects. **(1)** PCV is aware of the significance of finding a high-quality voting set, and proposes a progressive voter mining solution. As validated in Figure 5, PCV achieves outstanding performance due to the mined convincing voters. **(2)** Because of the high-quality voting set, PCV adapts well with the simple $L_{2}$ distance constraint. Although this constraint is ambiguous sometimes [22], it holds strong robustness with respect to many common nuisances. This trait makes the compatibility check process robust as well under different challenging conditions.

### 4.6. Time Efficiency

To compare the efficiency performance of tested methods, we record the time costs of these methods when processing initial correspondence sets with different cardinalities. This process is repeated for ten times, and the mean values are retained. The results are reported in Table 4.

Three main observations can be made from the figure. First, NN and NNSR can deal with thousands of correspondences in real-time. Nonetheless, both methods are sensitive to a number of nuisances. Second, ST is the most time-consuming method. Its time cost is significantly greater than others’ with dense initial correspondences. It is because the eigenvalue decomposition operation of ST is very time-consuming for high-order matrices. Third, CV is the most efficient one among three tested voting-based methods, followed by our PCV. Note that PCV obtains better feature matching performance than CV with respect to a number of nuisances. Overall, PCV is still a good option for applications with a strict demand on time consumption, because it can handle ∼1000 correspondences within 0.2 s.

### 4.7. Application to 3D Scene Registration

Feature matching usually plays a critical role in point cloud registration applications. Thus, we test PCV’s performance when applied to 3D scene registration. We conduct this experiment on the Aug_ICL-NUIM benchmark [46]. This benchmark assesses the registration performance via three metrics, i.e., precision, recall, and F-score (please refer to [46] for more details). Specifically, we perform 3D registration with a standard local feature matching-based pipeline proposed in [2]. This pipeline has been implemented in the point cloud library [47], and we dub the method as PCL. We propose a variant of PCL, namely PCL+PCV, which first selects the top-100 scored correspondences based on PCV and then feds them to the RANSAC estimator. The results are shown in Table 5.

**Figure 8:**
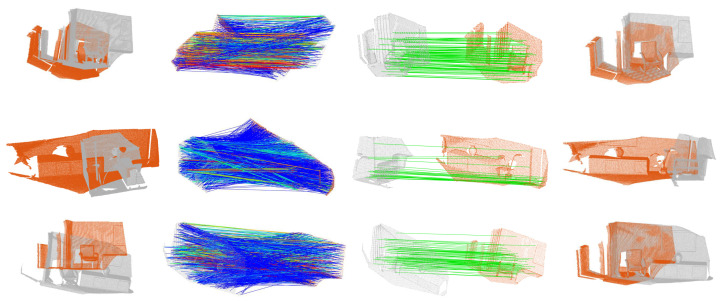
Sample registration results from the Aug_ICL-NUIM benchmark based on PCL+PCV. From left to right: initial point clouds, scoring results by PCV, top-scored correspondences selected by PCV, point clouds after registration.

It is clear that using PCV-selected correspondences can effectively boost the registration performance, as a gain of 11.4 percentages is achieved in terms of the F-score performance when performing PCV within the PCL framework. PCL+PCV also achieves the best F-score performance, the best recall performance, and the second best precision performance among all compared registration methods. This is reasonable because the correspondences selected by PCV are very consistent and RANSAC can estimate accurate transformations for correspondences sets with high inlier ratios. Note that PCL+PCV is slightly inferior to FGR in terms of precision. This is because FGR employs a more strict criterion for judging if a registration is correct. It results in better precision performance yet limited recall performance. Figure 8 presents some visual registration results obtained via PCL+PCV, indicating that consistent correspondences are generated by PCV and scene fragments are precisely aligned.

## 5. Conclusions

This paper has presented a robust feature matching method for 3D point clouds named PCV. The basic idea is to find a reliable voting set and assess the geometric consistency of each correspondence with the components in the voting set. Compared with existing methods, PCV holds two unique properties:

- PCV is the first method that highlights the significance of voting set definition for voting-based methods. It also affords a progressive and adaptive approach to mine convincing voters.
- Unlike existing methods that make great efforts to carefully design or combine geometric constraints, PCV is able to deliver outstanding performance with very simple constraints.

Feature matching experiments on three standard datasets and comparison with several state-of-the-art methods demonstrate that PCV is robust to noise, data decimation, clutter, occlusion, and data modality change. 3D registration experiments further show that PCV can improve the registration performance with existing pipelines.

Our PCV could be applied to the following application scenarios. First, 3D reconstruction from point clouds, which need to establish consistent point-to-point correspondences between point cloud views. Second, model-based 3D object recognition. This requires correspondences between the 3D model and the point cloud scene, which can be provided by PCV.

In the future, we plan to further improve PCV from the following two aspects. First, a better initialization could be achieved for PCV to determine the voting set, such as using existing feature matchers (e.g., GTM [36]). Second, an automatic stopping criterion for PCV iterations is desired, because correspondence sets with different inlier ratios may need different iterations.

## Figures and Tables

**Table 1:** The ROI performance (%) of PCV when using different initial voting sets.

$C_{\mathrm{voting}}^{0}$	K=20	K=50	K=100	K=500
NNSR	11.72	27.74	49.12	96.50
NN	11.25	27.74	47.96	94.20
Random	9.91	22.53	45.26	91.08

**Table 2:** The ROI performance (%) of PCV when using different geometric constraints.

Constraints	K=20	K=50	K=100	K=500
$L_{2}$	11.72	27.74	49.12	96.50
$L_{2}$ + Normal	12.05	27.74	50.02	96.61
$L_{2}$ + LRF	11.27	26.40	48.49	95.43
$L_{2}$ + Normal + LRF	11.90	27.58	49.40	96.50

**Table 3:** Correspondence grouping performance of tested methods on the U3OR dataset.

	Precision (%)	Recall (%)	F-Score (%)
NN	8.2	50.3	14.1
NNSR	20.8	47.3	14.5
ST [23]	14.5	30.1	19.6
SI [21]	22.0	50.8	30.7
CV [22]	30.2	68.4	41.9
LPM [33]	21.4	**73.2**	33.1
PCV	**31.8**	72.9	**44.3**

**Table 4:** Time costs (measured by second) of six feature matching methods respecting different numbers of initial correspondences. The symbol “−” denotes the time cost smaller than 1.0 × $10^{-4}$ s.

# Correspondences	NN	NNSR	ST	SI	CV	PCV
50	−	−	0.043	0.055	0.001	0.002
100	−	−	0.444	0.259	0.004	0.010
200	−	−	1.648	0.612	0.015	0.038
500	0.00010	0.00010	9.934	0.856	0.037	0.081
1000	0.00013	0.00013	39.955	1.159	0.074	0.182
2000	0.00018	0.00018	157.358	1.814	0.146	0.388

**Table 5:** 3D scene registration performance on the Aug_ICL-NUIM benchmark [46].

	Precision (%)	Recall (%)	F-Score (%)
OpenCV [39]	1.6	5.3	2.5
Super 4PCS [48]	10.4	17.8	13.1
PCL [2]	14.0	44.9	21.3
FGR [49]	**23.2**	51.1	31.9
CZK [46]	19.6	59.2	29.4
Ours (PCL + PCV)	21.8	**65.2**	**32.7**
